# Lipschütz Ulcers In 12-year-old Premenarchal Female Days After A Gastrointestinal Illness: A Case Report

**DOI:** 10.5811/cpcem.25343

**Published:** 2025-02-15

**Authors:** Kathleen Skeens, Laura Walker

**Affiliations:** * Mayo Clinic School of Graduate Medical Education, Department of Emergency Medicine, Rochester, Minnesota; † Mayo Clinic, Department of Emergency Medicine, Rochester, Minnesota

**Keywords:** Acute genital ulcers, gynecology, emergency medicine, Lipschütz ulcer, case report

## Abstract

**Introduction:**

Lipschütz ulcers are a rare immune-mediated reaction that commonly occurs in premenarchal females, usually associated with a recent viral illness.^1^ The treatment for Lipschütz ulcers consists of pain relief, topical steroids, and, in severe cases, a course of systemic steroids.^1^ A thorough history and exam, as well as an appropriate workup to rule out other causes of vaginal ulceration, should be completed.^2^

**Case Report:**

A premenarchal, 12-year-old female presented to the emergency department (ED) with her mother due to significant vulvar pain. Two days prior, the patient had a gastrointestinal illness associated with vomiting, diarrhea, and fever. On exam, she had significant swelling of the labia minor, discoloration with a necrotic appearance of the introitus, and brown vaginal discharge. The patient denied sexual intercourse, concern for retained vaginal foreign body, or vaginal trauma. Gynecology suggested the diagnosis of a rare post-viral immune-mediated reaction causing acute genital ulcerations, also known as Lipschütz ulcers. The patient’s treatment regimen included topical and systemic steroids, enteral opioid pain medication, and topical lidocaine. Her symptoms had resolved at her two-month follow-up visit.

**Conclusion:**

In summary, this case report discusses a previously healthy 12-year-old premenarchal female who presented to the ED due to vulvar swelling, pain, and vaginal discharge in the setting of a recent viral gastrointestinal illness. The patient was seen in the ED by gynecology and diagnosed with Lipschütz ulcers. Lipschütz ulcers are an uncommon condition causing acute genital ulcers.

## INTRODUCTION

Lipschütz ulcers are painful genital ulcers with unknown etiology. These ulcers present on the vulva and are often associated with recent bacterial or viral infections in adolescent females.^3^ Due to the severity and acuity of the symptoms, the patients who experience these acute genital ulcers may present to the emergency department (ED), as did the patient described in this case report. The limited number of publications available within emergency medicine literature has led to a lack of awareness of this disease by emergency physicians and therefore may lead to underdiagnosis in the emergency department setting. There is a need for emergency physicians to be aware of this disease to limit unnecessary testing and inaccurate treatments.^4^

## CASE REPORT

A 12-year-old premenarchal female presented to the ED with her mother due to vulvar pain and swelling. She was previously healthy, fully immunized for age, actively involved in volleyball, and had an average body mass index for age. Two days prior to presenting to the ED, she experienced vomiting, diarrhea, and fever associated with gastrointestinal illness, suspected to be viral in nature, which had since resolved. She denied respiratory symptoms. The patient had trialed home treatment with acetaminophen, ibuprofen, and warm baths to help the vulvar swelling and pain, but these interventions did not improve the swelling, pain, or discomfort. This was the patient’s first presentation for evaluation of this concern, and she had no prior evaluations by gynecology. She was premenarchal and denied sexual intercourse, a history of sexually transmitted infection, vaginal trauma, concern for retained foreign objects in the vagina, sexual trauma, or sexual assault.

On initial physician exam, the patient was afebrile, hemodynamically stable, well appearing, without increased work of breathing, however she was sitting very still in the hospital bed. The patient had mildly diffuse abdominal tenderness during the exam. On vaginal exam, there was swelling of the labia majora (left greater than right) with purple and necrotic ecchymosis, brown discoloration of the labia minor, and copious brown discharge flowing from the introitus. The patient had an initial workup in triage with negative swabs for SARS-CoV-2, influenza A, and influenza B. Broad workup was initiated and included a white blood cell count of 12 x 10^9^ per liter (L) (reference range: 3.8 – 10.4 x 10^9^/L) with a neutrophil count of 9.5 x 10^9^/L (1.5 – 6.5 x 10^9^/L), potassium of 3.5 millimoles (mmol)/L (3.6 – 5.2 mmol/L), sodium of 133 mmol/L (135 – 145 mmol/L), chloride of 99 mmol/L (102 – 112 mmol/L), creatinine of 0.79 milligrams per deciliter (mg/dL) (0.35 – 0.86 mg/dL). Urine microscopy showed red blood cells and bacteria, with a negative bacterial urine culture. Vaginal swabs were also collected in the ED and were negative for Chlamydia, gonorrhea, trichomonas, and herpes simplex virus (HSV). There was no growth on bacterial and fungal cultures.

Gynecology was consulted and upon examination they made the diagnosis of acute genital ulceration (Lipschütz ulcer). She was started on pain management with scheduled acetaminophen and ibuprofen. The patient was instructed to apply ice packs for twenty minutes once every hour to the area, topical clobetasol ointment two times daily, and topical lidocaine gel as needed. She was prescribed oral oxycodone. She was discharged from the ED with a follow-up visit with gynecology the following week.

At the follow up visit, gynecology started the patient on an oral prednisone taper, in addition to the topical steroids, given that the ulcers were still present and significant, although improved. She stopped using the clobetasol ointment after this visit due to pain with the application. She had another follow up with gynecology two weeks later and at that visit it was documented that her ulcers and pain had greatly improved. There were no concerns for a superimposed infection from appearance of the ulcers during the exam. The patient was instructed to follow up with her family medicine physician and discussion occurred regarding the increased likelihood of recurrence during times of stress, especially after viral infection or illness. It was explained that there would be no impact on her future fertility and no additional gynecology follow-up was warranted. The patient was prescribed topical lidocaine, topical clobetasol, and prednisone taper, if she experienced a flare in the future. The ulcers fully resolved at a follow up appointment with her primary care physician the following week.

**CPC-EM Capsule**

What do we already know about this clinical entity?

*Lipschütz ulcers are painful, necrotic ulcers due to an immune-mediated reaction in premenarchal females, usually associated with a recent viral illness*.

What makes this presentation of disease reportable?

*The presentation of Lipschütz ulcers in a pediatric emergency department in the setting of recent gastrointestinal illness*.

What is the major learning point?

*Lipschütz ulcers are painful, necrotic ulcers that can occur after a recent viral illness. They are usually self limited, however they may require treatment with topical or systemic steroids*.

How might this improve emergency medicine practice?

*Emergency medicine physicians should be knowledgeable about this uncommon diagnosis to prevent unnecessary workups and incorrect treatments*.

## DISCUSSION

This case report highlights the importance of emergency physicians considering the diagnosis of Lipschütz ulcers in adolescent females who present with acute genital ulcers after a recent viral illness to prevent invasive unnecessary workups and ineffective treatments. Lipschütz ulcers are described in the literature after a viral or bacterial infection, including influenza, streptococcal pharyngitis, with limited case reports describing the presentation after the COVID-19 vaccination and infection.^5^–^7^ Body aches, fatigue, and other nonspecific symptoms can occur during the presentation of the ulcers and should be a diagnosis of exclusion.^2^ Therefore, it is essential to take a thorough history and do a complete physical exam, along with any necessary workup to rule out other causes of acute genital ulcers, such as HSV, syphilis, chancroid, lymphogranuloma venereum, trauma, Crohn disease, psoriasis, trauma, fixed drug eruption, and Behçet syndrome.^1^,^3^

The ulcers are often described as bilateral “kissing lesions” that are painful, necrotic, with grayish exudate and signs of inflammation.^2^,^7^ Lipschütz ulcers have been reported in children but are more commonly reported in adolescents and young adults. The ulcers are often bigger than HSV lesions and painful, as compared to the non-painful chancre of syphilis. There is discussion in the literature regarding diagnostic criteria, however further research is needed regarding the utility of using this criteria in the ED.^3^ There is no role in biopsy for diagnosis, as case reports describe finding nonspecific inflammatory cell infiltration on biopsy.^6^,^8^ Resolution of ulcers occurs over the course of two to six weeks, and they may recur, especially during times of stress and viral illnesses.^7^,^8^

This is an uncommon condition with limited literature, specifically from an emergency physician perspective, which may lead to the underdiagnosis of this condition when patients present to the ED. Patients and parents should be counseled that there is no evidence to suggest a correlation between Lipschütz ulcers and sexually transmitted infections.^8^ Treatment is supportive with pain management, and the condition is self-limited. However, severe cases could be treated with topical corticosteroids with additional consideration for the use of systemic steroids.^1^,^2^,^6^ More literature is needed regarding the use of steroids for symptom management in this self-limited condition.

## CONCLUSION

In summary, this case report discusses the initial presentation of Lipschütz ulcers in a previously healthy 12-year-old premenarchal female who presented to the ED with her mother due to vulvar pain and swelling after a recent gastrointestinal illness. On vaginal exam, there was swelling of the labia majora (left greater than right) with purple appearing ecchymosis, brown discoloration of the labia minor, and brown discharger flowing from the introitus. The diagnosis of Lipschütz ulcer is uncommon; however, it should be considered in premenarchal females who deny sexual activity and have vulvar and vaginal ulceration on exam, especially in the setting of a recent viral illness. Misdiagnosis delays appropriate intervention and limit unnecessary workup. It is essential that emergency physicians make the correct diagnosis and arrange appropriate follow-up appointments with gynecology or primary care.

## References

[1] Moise A, Nervo P, Doyen J (2018). Ulcer of Lipschutz, a rare and unknown cause of genital ulceration. Facts, Views & Vis ObGyn.

[2] Mourinha V, Costa S, Urzal C (2016). Lipschütz ulcers: uncommon diagnosis of vulvar ulcerations. BMJ Case Rep.

[3] Sadoghi B, Stary G, Wolf P (2020). Ulcus vulvae acutum Lipschütz: a systematic literature review and a diagnostic and therapeutic algorithm. J Eur Acad Dermatol Venereol.

[4] DAG, Teixeira EPP, Lopes ACM (2021). Lipschütz Ulcer: An Unusual Diagnosis that Should Not be Neglected. Rev Bras Ginecol Obstet.

[5] Wojcicki AV, O’Flynn O’Brien KL (2021). Vulvar Aphthous Ulcer in an Adolescent After Pfizer-BioNTech (BNT162b2) COVID-19 Vaccination. J Pediatr Adolesc Gynecol.

[6] Sidbury R, Post TW (2024). Acute genital ulceration (Lipschütz ulcer). UpToDate.

[7] Lehman JS, Bruce AJ, Wetter DA (2010). Reactive nonsexually related acute genital ulcers: Review of cases evaluated at Mayo Clinic. J Am Acad Dermatol.

[8] Gay C, Kihara C, Haley A Lipschütz Ulcers: Classic Presentation of an Uncommon Condition. *Cureus*.

